# Area-Selective Atomic Layer Deposition of Ru Using Carbonyl-Based Precursor and Oxygen Co-Reactant: Understanding Defect Formation Mechanisms

**DOI:** 10.3390/nano14141212

**Published:** 2024-07-16

**Authors:** Jayant Kumar Lodha, Johan Meersschaut, Mattia Pasquali, Hans Billington, Stefan De Gendt, Silvia Armini

**Affiliations:** 1 Department of Chemistry, Faculty of Science, KU Leuven, B-3001 Leuven, Belgium; 2 Semiconductor Technology and System, Imec, Kapeldreef 75, B-3001 Leuven, Belgium

**Keywords:** self-assembled monolayer, defect analysis, Ruthenium ALD, area selective deposition

## Abstract

Area selective deposition (ASD) is a promising IC fabrication technique to address misalignment issues arising in a top–down litho-etch patterning approach. ASD can enable resist tone inversion and bottom–up metallization, such as via prefill. It is achieved by promoting selective growth in the growth area (GA) while passivating the non-growth area (NGA). Nevertheless, preventing undesired particles and defect growth on the NGA is still a hurdle. This work shows the selectivity of Ru films by passivating the Si oxide NGA with self-assembled monolayers (SAMs) and small molecule inhibitors (SMIs). Ru films are deposited on the TiN GA using a metal-organic precursor tricarbonyl (trimethylenemethane) ruthenium (Ru TMM(CO)_3_) and O_2_ as a co-reactant by atomic layer deposition (ALD). This produces smooth Ru films (<0.1 nm RMS roughness) with a growth per cycle (GPC) of 1.6 Å/cycle. Minimizing the oxygen co-reactant dose is necessary to improve the ASD process selectivity due to the limited stability of the organic molecule and high reactivity of the ALD precursor, still allowing a Ru GPC of 0.95 Å/cycle. This work sheds light on Ru defect generation mechanisms on passivated areas from the detailed analysis of particle growth, coverage, and density as a function of ALD cycles. Finally, an optimized ASD of Ru is demonstrated on TiN/SiO_2_ 3D patterned structures using dimethyl amino trimethyl silane (DMA-TMS) as SMI.

## 1. Introduction

Conventional lithography faces various challenges in addressing the misalignment issues in multi-level patterned structures [1]. In order to keep up with Moore’s law [2] and address these challenges, the area-selective atomic layer deposition (AS-ALD) bottom–up approach has emerged as a groundbreaking technique [1,3,4]. Along with the misalignment issue, ASD can mitigate the challenges associated with multiple lithography, deposition and etching steps in conventional top–down approaches [5,6,7,8]. One of the ways to achieve ASD is by modifying the local chemistry of growth and non-growth areas on a predefined patterned surface in such a way that the ALD precursor has an exclusive affinity to bond with the growth surface. This type of inherent selectivity has extensively been demonstrated using patterned Si surfaces with H and OH-terminal groups. Typically, the ALD of metal oxides has more affinity to OH-terminated surfaces while exhibiting a nucleation delay on H-terminated surfaces [9,10,11,12]. Another way to achieve selectivity is by deactivating the non-growth area towards ALD nucleation using polymers [13,14,15,16] or organic molecules such as self-assembled monolayers [17,18,19,20] (SAMs) or small molecule inhibitors [21,22,23] (SMIs). Area deactivation for AS-ALD by both SAMs and SMIs has been successfully demonstrated by many researchers, showing the pros and cons of SAMs vs. SMIs. SAMs are long-chain alkyl molecules with a head functional group that gets chemisorbed on the surface, and the alkyl chain relies on van der Waals interaction with adjacent groups to provide good inhibition [24,25]. SAMs can be deposited on surfaces by both immersion and vapor dosing, where immersion techniques take a longer time to form a film on the surface [26]. SMIs, on the other hand, due to their short chain, can be deposited on the surface much faster. The blocking capability of both organic films depends on the type of surface used, the thermal stability of the passivation layer, ALD conditions, etc. Depending on the stability of the passivation layer, the nucleation of unwanted deposits (e.g., nanoparticles) can start after certain ALD cycles in non-growth areas, disrupting the selectivity. A selectivity window can be calculated when defects are generated on the passivation layer, which refers to the number of ALD cycles before ALD material starts to grow in an area. Typically, ASD targets 90% selectivity at the target thickness, although this may vary depending on specific applications. The regeneration of organic molecules, selective etching, and ALD supercycles can be used to improve this selectivity window [3,27]. This paper covers Ru AS-ALD using both SAMs and SMI as inhibitors.

The ASD of Ru is of particular interest for its applications as a hard mask layer, alternative metal in interconnects and electrode materials. Ru has very high etch selectivity, making it promising as a hard mask layer or for patterning tone inversion [28]. It has a high work function (4.7 eV), low resistivity, good electromigration performance, chemical inertness, and dry etch susceptibility. Along with these properties, it has the potential to replace Cu in small dimension (below ~10 nm) nano-interconnects as it eliminates the need for a barrier layer and liner [21,29,30,31,32]. Furthermore, the seed layer for electroplating, electrode in dynamic random-access memory (DRAM), and gate in transistors can be other applications of Ru [33,34,35,36,37]. Various metal-organic precursors have been used for ALD of Ru, and some of them are summarized in Table 1.

As can be seen from Table 1, many precursors suffer the disadvantage of having low growth per cycle, high incubation cycles, producing films with high resistivity, and high deposition temperature etc. Therefore, developing an ALD Ru process that shows acceptable parameters is a critical requirement for industry. Ru (TMM) (CO)_3_ precursor offers a good trade-off between all these aspects. This metal-organic precursor has smaller organic ligands, making it more favorable to cover wider surface areas of the substrate, thereby increasing the growth per cycle to 1.5–1.7 Å/cycle, exceeding most of the existing precursors. Ru films formed using this precursor also have very low resistivity (13 µΩ.cm), meeting the criteria for nano-interconnects with shorter incubation cycles. This precursor is promising from an ASD point of view, as ALD is achieved at low temperatures. Most of the organic inhibitors are not stable at temperatures higher than 350 °C. Additionally, higher growth per cycle can be beneficial for selectivity as the required ASD thickness in the growth area can be achieved at early ALD stages. This reduces the exposure of the organic passivation to high temperatures or oxidative environments for a prolonged time. The high vapor pressure of this precursor also eliminates the need for additional precursor heating. As per the author’s knowledge, ALD growth on different substrates using this precursor has been reported before [29,30,55], but a detailed defect analysis using silane organic molecules as passivation agents and ASD on 3D patterns has never been conducted.

The main objective of this work is to develop and optimize a Ru ALD process utilizing Ru (TMM)CO_3_ precursor and O_2_ as a co-reactant to produce high-quality ASD films on TiN while minimizing defects on the SiO_2_ non-growth area. We evaluate the selectivity of the Ru ALD process on Si substrates coated with various self-assembled monolayers (SAMs), including octyl trimethoxy silane (TMOS), octadecyl trimethoxy silane (TMODS), 1H-1H-2H-2H-perfluorodecyl trimethoxysilane (FDTMS), as well as dimethylamino trimethylsilane (DMA-TMS) SMI. The TMOS SAMs are deposited using both vapor phase and immersion techniques to compare their effectiveness as passivation layers. These organic molecules belong to the silane group, encompassing a range of chain lengths from as long as C18 to as short as C1. Moreover, our investigation includes defect analysis, focusing on the nucleation, diffusion, and coalescence of Ru nanoparticles on the passivated non-growth area. We delve into defect coverage, density, and particle size distribution. Additionally, we demonstrate the ASD of Ru on 3D structures etched in SiO_2_ and landing on TiN, where the former represents the non-growth area, while the latter serves as the growth area. Therefore, Ru is selectively deposited on the TiN surface.

## 2. Materials and Methods

Materials: 300 mm silicon wafers were provided by Sun-Edison semiconductors. Analytical grade (99.9% purity) toluene was purchased from Fischer Chemical (Brussel, Belgium), and tri-methoxy octadecyl silane (TMODS), tri-methoxy octyl silane (TMOS) and 1H-1H-2H-2H-perflourodecyl tri-methoxy silane (FDTMS) SAM pre-cursors were provided by Sigma-Aldrich (Hoeilaart, Belgium) and were used as supplied. Tricarbonyl (tri-methylene methane) ruthenium (Ru (TMM)CO_3_) was supplied by Tanaka Kikinzoku (Tokyo, Japan). The fabrication process of TiN/SiO_2_ 3D line/space pattern is discussed elsewhere [28].

Substrate preparation and characterization: 10 mM concentration of TMODS and TMOS SAM was deposited on Si surface by immersion technique using toluene as a solvent. The optimization condition can be found in a previous paper [18]. Vapor phase deposition of TMOS and FDTMS SAM was performed in a Heratherm OM180 over from ThermoScientific. TMOS SAM was deposited at a deposition temperature of 160 °C for 2 h, whereas FDTMS SAM was deposited at the same temperature for 1 h. The chamber pressure for both depositions varied between 9–13 mBar. DMA-TMS deposition was performed using a TEL Tactras tool, which has a thermal degas step followed by vapor deposition. The substrate was kept at 5 Torr N_2_ ambient in a closed reactor at 250 °C for 10 min to desorb residual moisture and organics. Afterward, the reactor was evacuated and filled with a mixture of 500 sccm DMA-TMS and 350 sccm N_2_ up to a pressure of 5 Torr. A saturation time of 300 s was reached [56]. Before SAM and SMI deposition, the Si surface was pre-treated with ultraviolet ozone (UVO_3_) for 15 min to remove the organic contaminants and to maximize surface hydroxyl groups.

Organic molecule quality was assessed using water contact angle (WCA) analysis. Static WCA has performed ex situ in the DataPhysics tool using de-ionized water. The tool is equipped with a camera from Teli and a 500 μL syringe from Hamilton. Contact angle measurements were performed using a sessile drop of 2 μL of water with a dispensing speed of 1 μL/s. WCA for liquid TMODS, liquid TMOS, vapor TMOS, vapor FDTMS and vapor DMA-TMS is 109°, 91°, 100°, 113.5° and 97°, respectively. Here, ”liquid” refers to deposition by immersion technique and “vapor” refers to deposition from the vapor phase.

ALD and ASD assessment: Rutherford backscattering spectroscopy (RBS) was used to measure the thickness of Ru films on the surface. It uses a He^+^ beam ion with an ion energy of 1.523 MeV with scattering angle and sample tilt of 170° and 11°, respectively. A 1 × 1 mm^2^ beam spot size was used. The equivalent film thickness of Ru was derived from the areal density calculated by RBS. The assumption is that a Ru mass density of 12.36 g/cm^3^ corresponds to an atomic density of 7.37 × 10^22^ Ru atoms/cm^3^. The selectivity (S) of the deposited material is calculated by Equation (1).

(1) $$S=\frac{\theta_{G}-\theta_{NG}}{\theta_{G}+\theta_{NG}}$$

where, $\theta_{G}$ and $\theta_{NG}$ are the amount of material deposited in the growth area and non-growth area, respectively.

The ALD of Ru was carried out in a Veeco Savannah S300 crossflow reactor under a N_2_ atmosphere. ALD was carried out with a sequential pulse of Ru (TMM)CO_3_ precursor using O_2_ as a co-reactant. The Ru (TMM)(CO)_3_ canister was placed at 35 °C because of its high vapor pressure and pulse time, which varied between 5 s–and 14 s. An N_2_ flow of 50 sccm was employed to deliver the precursor to the reactor, resulting in a precursor pressure of 0.3–0.4 Torr. O_2_ flow was set to 1 Torr with pulse time varying between 0.5 s–13 s. A 20 s of N_2_ purging was applied after each dose of precursor and co-reactant. The ALD was carried out with a reactor temperature between 180–220 °C. ALD was performed on sample coupons with 3 × 3 cm in dimension. Atomic force microscopy (AFM) measurements were performed using the Bruker dimension edge tool provided with the Olympus OMCL-AC160TS-R3 probe. The AFM was used to measure the surface roughness and topography of Ru films using a scan area of (2 × 2) µm. Scanning electron microscopy (SEM) was performed using Helios NanoLab 460HP FEI SEM tool using a through-the-lens detector in secondary electron mode with a 3 kV applied potential and a probe current of 0.10 nA. SEM measurements were done to observe the morphology of the Ru surface and defects in non-growth areas. Top–down SEM (TD-SEM) imaging was performed with a 0° tilt angle with a surface normal for the planar substrate, whereas cross-section SEM (X-SEM) was performed to measure the Ru thickness and uniformity throughout both the planar and 3D surface. A SEM image at a 20° tilt angle was done to see the Ru defectivity on the SiO_2_ wall on the 3D surface. ImageJ software (v 1.52a) was used to adjust the contrast in SEM images and to extract the defectivity in the passivated area. The analysis excludes any particle with less than 1 nm radius (resolution limit) and includes edge particles. The defectivity in the passivated area has been defined by three parameters in this work, namely defect coverage ($C_{def}$), average defect density ($\rho_{def}$), and average defect radius ($r_{avg}$).

(2) $$C_{def}=\frac{A_{def}}{A_{p}}*100$$

where, $C_{def}$ (in percentage) is the ratio of $A_{def}$ The total defect area and $A_{p}$ is the area covered by Ru defects in the passivated region multiplied by 100.

Average defect density ($\rho_{def}$) is the number of nanoparticles seen in a SEM image per unit area and is defined as ratio $N_{def}$ and $A_{p}$.

(3) $$\rho_{def}=\frac{N_{def}}{A_{p}}$$

The above calculations were done by using MATLAB version R2021a. It was also used to calculate the probability density of particles. Cross-sectional transmission electron microscopy (TEM) combined with energy dispersive X-ray spectroscopy (EDS) was measured to assess the quality and selectivity of Ru films on the TiN surface and to see defects of Ru on SiO_2_ surface in 45 nm line/space 3D structure. The cross-section was done on a FEI Nanolab 450HP dual beam focused ion beam (FIB) tool, and TEM images/EDS maps were collected using the FEI Titan G2 60–300 tool. Prior to FIB, the sample was coated with a thin e-beam Pt layer followed by a drop-casted organic layer (spin-on-carbon) baked at 150 °C and then an ion beam deposited Pt layer. The cross-section was created by milling a trench at 30 kV in the region of interest. The sample was attached to the TEM grid and initially thinned at 30 kV, and the final process was performed at 5 kV. Ru etch was carried out in LAM Versys chamber at 60 °C using zero-bias O_2_/Cl_2_ plasma chemistry.

## 3. Results & Discussion

### 3.1. ALD of Ruthenium

**Figure 1:**
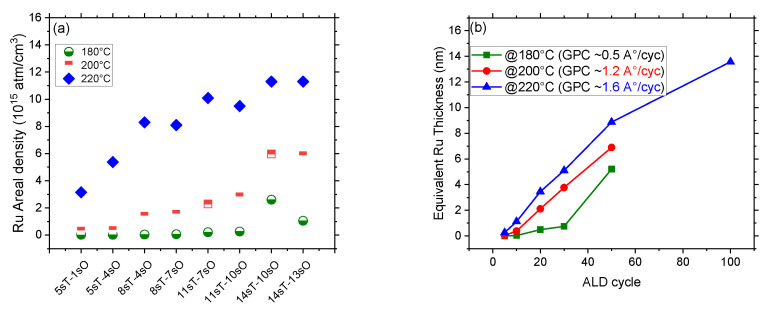
(**a**) RBS Ru areal density after 10 ALD cycles at three different temperatures (180, 200, and 220 °C) and different precursor and co-reactant pulse times (here, T denotes Ru precursor pulse time whereas O is oxygen pulse time). (**b**) The equivalent Ru thickness vs. ALD cycles at different temperatures.

The Ru ALD process is studied on homogenous silicon substrates with native oxide, which were cleaned with a 15-min UV-ozone surface. The tested ALD temperatures are 180 °C, 200 °C and 220 °C. Higher temperatures are not tested due to the thermal degradation of both the surface organic inhibitors and the ALD precursor [30]. Ru precursor exposure time varies between 5 s to 14 s, and that of oxygen co-reactant is between 0.5 s and 13 s. The initial focus of the experiment is to optimize the ALD conditions. Figure 1a shows the Ru areal density measured by RBS after 10 ALD cycles carried out at different temperatures and pulse times.

The graphs clearly show that the Ru areal density is much higher if the ALD temperature is set at 220 °C. Also, the precursor and co-reactant pulse times have quite a significant effect on the amount of Ru deposited. The lower the precursor and co-reactant pulse times, the lower the Ru GPC, whereas the GPC reaches a plateau beyond 11 s and 7 s of precursor and oxygen pulse times, respectively. The plateau of Ru areal density is found at an equivalent Ru surface concentration of ~10.5 × 10^15^ atm/cm^2^. Figure 1b shows the equivalent Ru thickness as a function of ALD cycles. Ru ALD is performed in the T-range between 180–220 °C in steps of 20 °C using a fixed precursor and co-reactant pulse times of 11 s and 10 s, respectively. The GPCs are calculated to be 0.5, 1.2, 1.6 Å/cycle at 180 °C, 200 °C, and 220 °C process temperatures, respectively. As expected, the GPC is found to be lower at 180 °C when compared to the GPC at 220 °C. The growth of Ru above 200 °C is linear with the number of performed ALD cycles, whereas, below 200 °C, there is a delay of about 10 cycles before the GPC increases significantly.

Based on the GPC considerations, a temperature of 220 °C is selected for further ALD experiments. The Ru films deposited at this temperature exhibit a surface roughness of 0.05 nm, 0.06 nm, and 0.09 nm after 10, 30, and 50 ALD cycles, respectively (see Supplementary Information S1). The equivalent Ru thickness observed from RBS after 10, 30, and 50 ALD cycles is 1.2, 5.1, and 8.9 nm, respectively. TD-SEM and X-SEM imaging were done to check the morphology and uniformity of the film (see Supplementary Information S2). The SEM images show that the Ru film is not coalesced after 10 ALD cycles, whereas a continuous film is achieved after 30 ALD cycles. The Ru thicknesses calculated from the X-SEM images are 2.1, 5.9, and 10 nm for 10, 30, and 50 ALD cycles, respectively, which are in agreement with the thicknesses extracted from the RBS measurements assuming a Ru density of 12.36 g/cm^3^. In light of the observed GPC of Ru, which is significantly higher than the theoretical monolayer growth rate for Ru on the Ru(0001) surface, it is possible that our process exhibits characteristics of both ALD and chemical vapor deposition (CVD) under the experimental conditions employed. In addition, density might be lower than high-temperature Ru ALD due to C and O incorporation. The apparent DC resistivity of 4 ohm-cm in an ~8 nm thick film is recorded, which strongly suggests the presence of impurities such as carbon (C) and oxygen (O) within the film. The study further focuses exclusively on its application as a hard mask layer. Its unsuitability for nano-interconnects or electrode material applications is underscored by this highly apparent DC resistivity.

### 3.2. Selectivity and Defect Analysis

Optimized Ru ALD: The selectivity of the Ru ALD process is studied using an organic inhibition coating. Since trifluoromethyl-terminated SAMs show a higher water contact angle (113.5°) with respect to methyl-terminated SAMs, Ru selectivity is tested on blanket Si substrates coated with a SAM derived from an FDTMS precursor. Since the Ru precursor has polar ligands, a hydrophobic surface with a high-water contact angle is expected to be less favorable with respect to physisorption and chemisorption. The TD-SEM images of the FDTMS functionalized oxidized Si substrates after 5 to 50 Ru ALD cycles are reported in Supplementary Information S3. Ru defects on the FDTMS passivated surface are already observed after 5 ALD cycles, while above 30 ALD cycles, the Ru nanoparticles coalesce, leading to the formation of a continuous Ru film.

**Figure 2:**
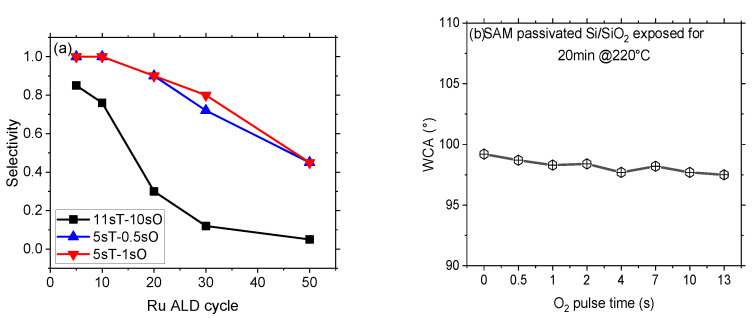
(**a**) Selectivity of Ru on FDTMS passivated Si surface and pristine Si surface for 5 to 50 ALD cycles. (**b**) WCA change is associated with the TMOS passivated Si surface when exposed at 220 °C to 20 min oxygen co-reactant, varying the oxygen pulse times.

The selectivity of the Ru ALD process on the FDTMS passivated Si surface is calculated according to Equation (1), considering the FDTMS passivated Si surface as a non-growth area and Si surface as a growth area and plotted in Figure 2a as a function of the number of performed ALD cycles. A selectivity of 0.85 is observed after 5 ALD cycles, while it drastically drops to 0.3 after 20 ALD cycles. Our goal is to achieve a selectivity higher than 0.9 at the target ASD Ru thickness of 2–3 nm, assuming the Ru film is continuous. This selectivity drop can be attributed to the reduced SAM’s thermal stability at 220 °C and/or to the high reactivity of the Ru precursor in the presence of oxygen co-reactant degrading the passivation layer.

To investigate the stability of the SAM at 220 °C in the presence of oxygen, the SAM passivated Si surface is exposed at 220 °C to oxygen with pulse times varying from 0.5 s to 13 s for a duration of 20 min. Changes in WCA are reported as a function of different oxygen pulse times, as shown in Figure 2b. The change in WCA is not significant even at the maximum oxygen pulse time of 13 s, where the WCA decrease is still limited to 1.7°. This shows that the SAM layer is thermally stable at a temperature of 220 °C in the presence of oxygen. The second probable reason for low selectivity can be due to the co-presence of Ru and oxygen. Ru is recognized as a thermal catalytic substance and, at high temperatures, is capable of dissociating oxygen to produce atomic oxygen [57,58,59]. The defects in the non-growth area can arise from the oxidative degradation of FDTMS SAM catalyzed by the first Ru nuclei. In this scenario, the ALD procedure employs a 10 s oxygen pulse duration, thereby increasing the availability of atomic oxygen and potentially compromising the integrity of the passivation layer.

**Figure 3:**
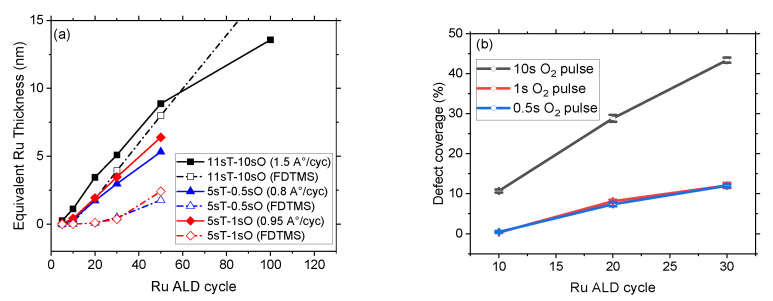
(**a**) Equivalent Ru thickness using different precursor and co-reactant pulse times on blanket Si substrates (solid points) and FDTMS passivated Si substrates (unfilled points). The T in the graph represents Ru precursor whereas O indicates oxygen co-reactant). (**b**) Ru defect coverage as a function of ALD cycles using 0.5 s, 1 s, and 10 s oxygen pulse times.

To ascertain the impact of the oxygen co-reactant in the presence of Ru on the passivation film, Ru ALD is performed with relatively low oxygen pulse times of 0.5 s and 1 s, whereas the Ru precursor pulse time is set to 5 s. Figure 3a shows the equivalent Ru thickness reported as a function of different co-reactant pulse times. Reducing the oxygen pulse times to 0.5 s and 1 s suppresses the formation of atomic oxygen and thereby inhibits Ru nanoparticle growth on FDTMS passivated Si surface, i.e., defects remain undetectable up to 20 ALD cycles. The calculated Ru selectivity (S) corresponding to 20 ALD cycles is >0.9 when using 0.5 and 1 s of oxygen pulse time, which is substantially higher than the selectivity value of 0.3, as reported in Figure 2a using 10 s oxygen pulse time. This points to a defectivity generation mechanism triggered by the Ru-induced oxidative deterioration of the SAM.

A Ru GPC of 1.6 Å/cycle (Figure 1b) was calculated when using the 10 s oxygen pulse time in the ALD process. This GPC decreased to 0.80 Å/cycle and 0.95 Å/cycle while using oxygen pulse times of 0.5 s and 1 s, respectively. Although this reduction in GPC is significant, the ALD process still holds an above-average deposition rate compared to most of the processes performed with other Ru precursors, as seen in Table 1. Figure 3b shows the Ru defects areal coverage as calculated from TD-SEM images (see Supplementary Information S4) on the FDTMS passivated Si surface after 10 to 30 ALD cycles. The Ru surface coverage after 30 ALD cycles with 0.5 s and 1 s oxygen pulse times is comparable to the defectivity observed after only 10 ALD cycles with 10 s oxygen pulse time. To summarize, the Ru ALD process consisting of 11 s precursor and 10 s oxygen pulse time at 220 °C shows high GPC on pristine Si surfaces but very low selectivity on FDTMS passivated Si surfaces, while 5 s precursor and 1 s oxygen pulse time led to lower GPC and improved selectivity (0.9 after 20 cycles).

**Figure 4:**
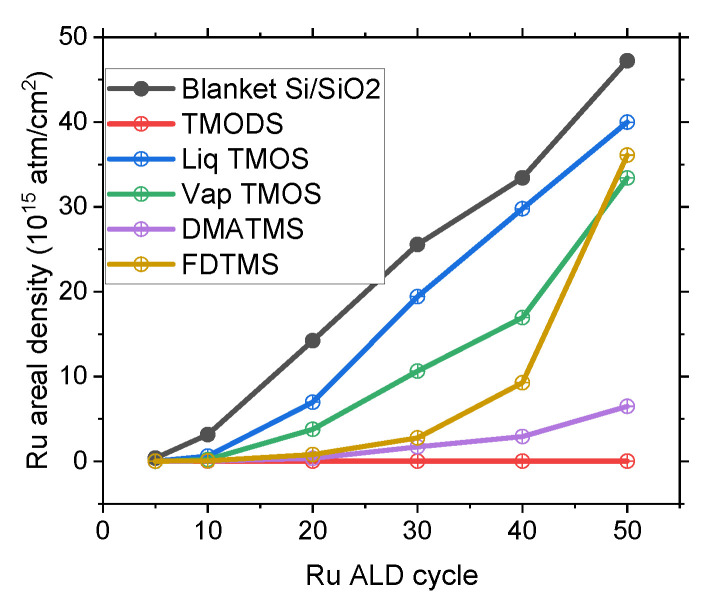
Ru Areal density calculated from RBS analysis on pristine Si and organic inhibitor passivated Si surfaces.

Selectivity and defect analysis with optimized Ru ALD process: In the scope of this work, to find a trade-off between GPC and selectivity of Ru on SAM passivated substrates, the latter condition is chosen for further investigations using alternative Si passivation. Ru selectivity is explored on (a) Si substrate passivated with TMODS deposited from solvent solution, (b) TMOS deposited from the vapor phase, (c) solvent solution, (d) DMATMS, and (e) FDTMS deposited from the vapor phase. Figure 4 shows the Ru areal density on Si passivated surfaces with different SAMs as a function of the number of performed ALD cycles. As our goal is to reach a selectivity higher than 0.9 for 2–3 nm of coalesced Ru film, the selectivity corresponding to more than 20 ALD cycles is analyzed to compare the effectiveness of the studied organic layers.

TMODS SAM passivated Si exhibits the largest Ru nucleation inhibition window, with the process selectivity being above 0.99 after 50 ALD cycles with a corresponding Ru areal density of 0.02 × 10^15^ atm/cm^2^. The high selectivity can be attributed to the presence of a long alkyl chain in the TMODS-derived SAM (18 C atoms), which favors the formation of a highly ordered organic layer due to interchain van der Waals interactions [60]. The high crystallinity of the SAM is responsible for its ALD inhibition capabilities [61]. TMOS deposited by either immersion or vapor phase techniques initially inhibits Ru growth up to 10 ALD cycles. However, upon 20 ALD cycles, RBS measurements on TMOS passivated surface reveal Ru areal density of 6.9 × 10^15^ atm/cm^2^ and 3.7 × 10^15^ atm/cm^2^ for TMOS deposited from the liquid and vapor, respectively. These measurements highlight poor Ru ALD selectivities of 0.34 and 0.60 for the TMOS blocking layer deposited from the liquid and vapor phases, respectively. The superior vapor phase TMOS inhibition is coherent with its higher WCA when compared to the WCA of TMOS deposited by immersion.

**Figure 5:**
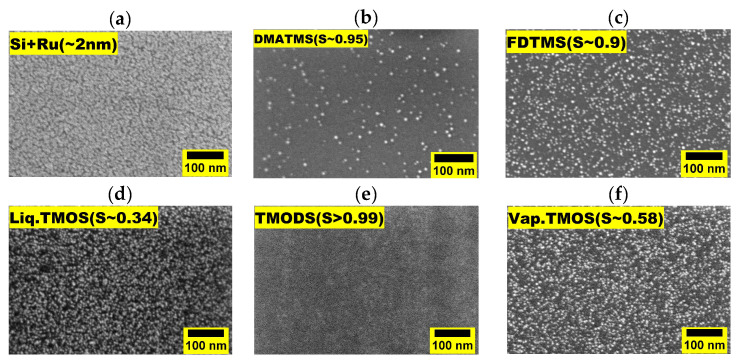
Top–down SEM images of (**a**) Si substrate; (**b**) DMATMS passivated Si; (**c**) FDTMS passivated Si; (**d**) Liquid TMOS passivated Si; (**e**) TMODS passivated Si; (**f**) Vapor TMOS passivated Si, after 20 Ru ALD cycles and their corresponding selectivity.

Finally, Si substrates functionalized with FDTMS and DMATMS and exposed to 20 ALD Ru cycles lead to selectivity of 0.95 and 0.90, respectively. Ru areal density on pristine Si and organic molecule passivated surface for 5 and 10 ALD cycles is shown in Supplementary Information S5. Figure 5 shows the TD-SEM images of Ru films on blanket Si substrate and organic layer passivated Si surfaces after 20 ALD cycles. Images of such substrates after different ALD cycles are reported in Supplementary Information S6. Overall, the selectivity inferred from the TD-SEM images is in line with the RBS results.

**Figure 6:**
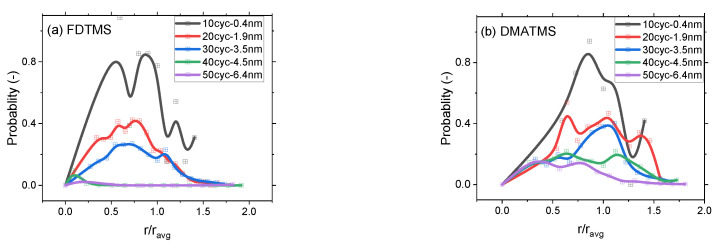

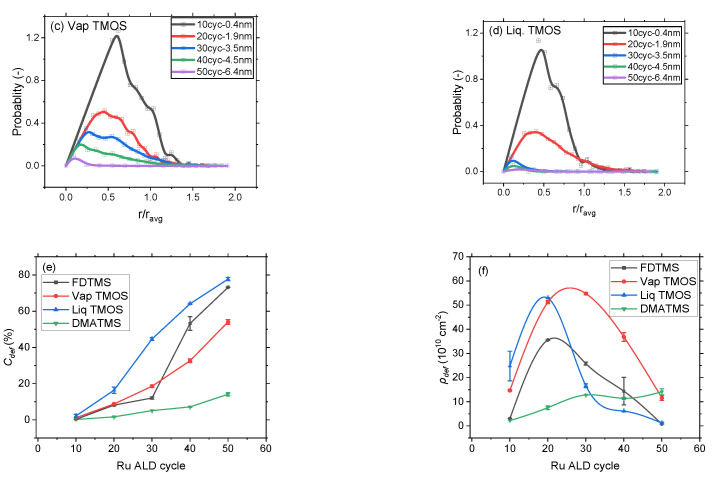
Probability density of Ru particle distribution on SAM passivated surface vs. the ratio of particle radius to the average radius (*r*/$r_{avg}$); (**a**) FDTMS; (**b**) DMATMS; (**c**) Vapor TMOS; (**d**) liquid TMOS. (**e**) Ru defect coverage ($C_{def}$) on SAMs passivated surface. (**f**) Average Ru defect density ($\rho_{def}$) on SAM passivated surface vs. ALD cycles.

The nucleation and evolution of defects on SAM and SMI passivated Si substrates are studied as samples undergo 10 to 50 Ru ALD cycles performed at 220 °C. The defectivity observed by SEM is quantified in terms of defect coverage ($C_{def}$) and average defect density ($\rho_{def}$) (Equations (2) and (3)), and the results are presented in Figure 6e,f as a function of the Ru ALD cycles. Figure 6a–d show the particle size distribution (PSD) of the Ru particles formed on the different organic blocking layers considered in this work as a function of the ratio between defect radius (*r*) and average defect radius ($r_{avg})$. The SEM images for each PSD data point are shown in Supplementary Information S6, and the particle average radius ${(r}_{avg})$ is calculated by taking the average of the particle radii derived from the SEM images. While decreasing the oxygen pulse duration during ALD enhances process selectivity, SEM examination reveals the substantial influence of different passivation layers on the Ru nanoparticles growth mechanism. The occurrence of Ru defects on passivated Si surfaces varies notably depending on the type of passivation layer employed in this study. Defect generation can arise for several reasons. An explanation could be that the Ru precursor diffuses through the organic passivation layer, reaching the underlying surface and thus initiating undesired nucleation. As the initial nuclei grow through the coalescence of Ru nanoparticles, defect coverage increases. Another defect formation mechanism consists in the physisorption of the Ru precursor on the surface of the organic passivation layer, which might be oxidized, thereby establishing nucleation sites for material deposition [62,63,64]. These mechanisms collectively contribute to the loss of selectivity.

In Figure 6e, while $C_{def}$ increases with the ALD cycles, it varies depending on the type of passivation layer. As an example, after 20 ALD cycles, the $C_{def}$ is 8.1% ± 0.4 and 8.7% ± 0.1 for surfaces passivated with FDTMS and Vapor TMOS SAM, respectively. However, a comparable $C_{def}$ of 7.1% ± 0.2 is observed for DMATMS passivated Si surface after 40 ALD cycles. In earlier studies, it was suggested that these defects might grow via a layer-by-layer mechanism [65]. However, when considering the DMATMS layer, the average defect particle radius after 20 cycles measures 2.6 nm, whereas layer-by-layer growth would imply an expected radius of 1.9 nm. This shows that surface diffusion and nanoparticle coalescence play a significant role in particle growth.

For each substrate, the mechanism governing $\rho_{def}$ and PSD is studied considering 10 to 50 Ru ALD cycles. Figure 6b shows the PSD probability as a function of *r*/$r_{avg}$ on DMATMS passivated Si surface. The ratio between the particle radius (*r)* and the average particle radius ($r_{avg})$ provides insight into the distribution of the particle sizes. If this ratio is higher than 1, it suggests that the majority of particles are larger than the average particle size, indicating a skewed distribution towards larger particles. Conversely, if the ratio is less than 1, it implies that the majority of the particles are smaller than the average size, indicating a skewed distribution towards smaller particles. A ratio of 1 indicates a uniform distribution where the particle sizes are evenly distributed around the average size.

In Figure 6b, we observe that after 10 ALD cycles, the probability of PSD is highest when *r*/$r_{avg}$ is close to 1, suggesting that Ru particles of comparable radius are evenly distributed on the passivated surface. The maximum probability is observed to shift towards *r*/$r_{avg}$ ≥ 1 for 20-to-40 ALD cycles, suggesting that larger particles than the average radius are predominant. This occurs when particle diffusion and coalescence are more likely to happen than when new particles are formed. The same can be inferred from the average defect density, which stays constant at (12.1 ± 0.9) × 10^10^ defects per cm^2^ between 30–40 ALD cycles suggesting an equilibrium between particle nucleation and diffusion [17]. In order to explain the observed mechanisms, considerations on the passivation layer can be made. Thanks to their small size, the densely packed trimethylsilyl groups form bonds with a considerable portion of the available hydroxyl groups on the Si surface, thereby preventing the diffusion and chemisorption of the Ru precursor to the underlying Si surface. The small amount of defects initially generated during the first ALD cycles could be attributed to the physisorption of the Ru precursor onto the surface of the DMATMS layer, followed by subsequent particle formations and coalescence.

TMOS SAM passivated Si surface exhibits significantly different behaviour from that of DMATMS passivated surface. Despite the longer chain length of TMOS, it demonstrates considerably lower blocking capability towards the formation of Ru defects. It is important to note that when the alkyl chain length is 8 or shorter, the energy balance is strongly changed since the chain-chain interactions can no longer compete with the head group-substrate interactions [66,67]. The reduced van der Waals interactions result in a disordered layer. Additionally, TMOS, while still providing steric hindrance, allows for a larger fraction of free hydroxyl groups on the Si surface when compared to DMATMS. These factors can facilitate the diffusion of Ru precursor to the underlying Si substrate, creating nucleation sites. Additionally, defects can be generated through physisorption as well.

Combining both factors, the TMOS passivated Si surface shows defect generation at low ALD cycles, leading to the coverage of over 20% of the surface within 20 ALD cycles. A lower number of defects is observed on TMOS deposited from the vapor phase than on TMOS deposited by immersion. Along with higher WCA, the former has a very stable SAM thickness of 0.77 ± 0.01 nm, while the latter shows lower uniformity depending on immersion time [18]. The calculated Ru $\rho_{def}$ on TMOS deposited by immersion is (52 ± 1.1) × 10^10^ per cm^2^ after 20 ALD cycles, and it drastically decreases to (16.6 ± 0.8) × 10^10^ per cm^2^ after 30 ALD cycles. In the case of vapor TMOS, the $\rho_{def}$ is (54 ± 1.1) × 10^10^ per cm^2^ after 30 ALD cycles and (36 ± 1.1) × 10^10^ per cm^2^ after 40 ALD cycles. The significant decrease in $\rho_{def}$ suggests that the nanoparticles have coalesced to such an extent that, rather than existing as discrete particles, they begin to behave like a continuous film. As shown in Figure 6c,d, the Ru PSD for 10, 20 and 30 ALD cycles on TMOS passivated Si surface shows the maximum probability in the region where *r*/$r_{avg}$ is close to 0.5, pointing to the formation of a significant number of small Ru particles. However, this probability value tends towards zero beyond 30 ALD cycles, suggesting that a Ru film is formed by coalescence. This trend in the probability of PSD for TMOS passivated surface is in line with the previously discussed $\rho_{def}$ trend. This suggests that defect formation and coalescence are occurring simultaneously. The formation of Ru defects on FDTMS passivated Si surfaces follows a similar trend as on TMOS passivated surfaces, but FDTMS shows better passivation capability. This difference in selectivity can be attributed to the fluorine molecule in the backbone of the FDTMS molecule, which helps suppress the physisorption of the Ru precursor on the SAM surface. However, the repulsion between adjacent fluorine atoms due to their electronegativity may still allow Ru precursors to diffuse and deposit on the Si surface underneath [68]. This suggests that surface diffusion might be the main cause of defect formation. The probability of PSD after 10 ALD cycles is highest when *r*/$r_{avg}$ is close to 1 (Figure 6a). However, the probability peak shifts towards *r*/$r_{avg}$ ≤ 1 after 20 and 30 ALD cycles, indicating the formation of smaller particles. Beyond 30 ALD cycles, this peak diminishes towards zero, indicating film formation and coalescence.

In summary, it is observed that the physisorption of Ru precursor is the main cause of defect formation on DMATMS passivated Si surfaces, while diffusion of precursor through the organic films to reach the underneath substrate is the main defect formation mechanism on FDTMS passivated surfaces. On TMOS passivated surfaces, Ru defects are formed by both diffusion and physisorption. The defect formation mechanisms are significantly influenced by the type of passivation, its ordering, thickness, chemical composition, and structure. These factors have a crucial impact on selectivity.

### 3.3. ASD of Ru on SiO_2_/TiN Pattern

**Figure 7:**
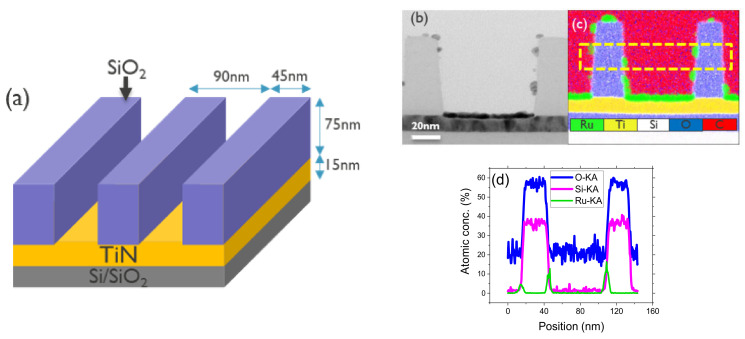
(**a**) Schematic of the 3D line/space TiN/SiO_2_ patterns. Forty Ru ALD cycles performed on DMATMS passivated TiN/SiO_2_ pattern at 220 °C; (**b**) Bright field-TEM image; (**c**) EDS mapping of Si, O, C, Ru, N, Ti; (**d**) line scan elemental profile from the highlighted area in (**c**) are presented showing Si, O, and Ru lines.

We investigate the ASD of Ru in 90 nm pitch 3D line/space patterns etched in 45 nm thick SiO_2_ landing on TiN at the bottom of the trenches, as shown in the schematic of Figure 7a. Leveraging the learning on SiO_2_ passivation, the ASD of Ru on TiN with respect to SiO_2_ is the goal of this ASD Ru study on patterned structures. In previous experiments, TMODS is identified as the most suitable layer for blocking Ru deposition on SiO_2_. However, TMODS cannot be applied to SiO_2_/TiN patterns because the SAM is grafted on both SiO_2_ and TiN, thereby inhibiting ASD on both materials. The WCA value measured on TMODS passivated pristine TiN surface, is 110° ± 0.3, demonstrating the lack of selectivity of TMODS between oxidized TiN and SiO_2_. DMATMS is selected for this study, as it can be selectively deposited on SiO_2_ [69].

**Figure 8:**
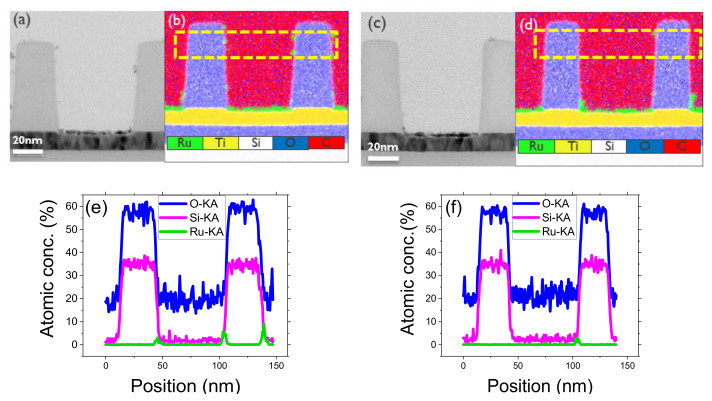
TEM and EDS analysis of 45 nm wide TiN/SiO_2_ line/space 3D pattern upon 40 ALD super cycles (left (**a**,**b**,**e**)) and 7 s etch after 40 ALD cycles (right (**c**,**d**,**f**)). BF image (**a**,**c**) and EDS spectral image (**b**,**d**) are presented with emissions from Si, O, C, Ru, N, and Ti elements. Line scan profiles (**e**,**f**) from the highlighted area in (**b**,**d**) are presented, showing Si, O, and Ru lines.

SEM, cross-sectional TEM, and EDS analysis are employed to evaluate the ASD of Ru on a 45 nm wide SiO_2_/TiN pattern. Ru ALD is performed after DMATMS passivation for 20, 30, 40, and 50 cycles. The corresponding SEM images are provided in Supplementary Information S7. The SEM images show a selective Ru deposition with defects on the SiO_2_ sidewall and top surface. Both the thickness of the selective Ru on the TiN growth area and the Ru defects on the SiO_2_ increase with each ALD cycle. The Ru GPC on TiN is lower compared to the GPC on the Si surface, which is in line with our results on the pristine TiN surface (refer to Supplementary Information S8). From the TEM image (Figure 7b), a Ru thickness of 4.4 ± 0.2 nm is observed on TiN after 40 ALD cycles, while on blanket TiN layers, the same ALD process leads to a Ru thickness of ~4.6 nm, which is notably comparable. EDS spectral images (Figure 7c) and elemental profile analysis (Figure 7d) were conducted to quantify the Ru defects on SiO_2_ walls, which were found to be as high as ~18% Ru atomic concentration after 40 ALD cycles. To resolve the defects in the non-growth area, two approaches were pursued. The first involved attempting ALD supercycles, where DMATMS re-passivation was performed after every 10 ALD cycles, totaling 40 cycles. The second approach was to etch the Ru defects formed after 40 ALD cycles. Figure 8a,b,e show the TEM image, EDS spectral and elemental line scan profile, respectively. An average thickness of 3.4 ± 0.2 nm of selective Ru is calculated from the TEM image, which is lower than the expected value of 4.4 ± 0.2 previously observed after 40 ALD cycles.

A potential explanation for the reduction in Ru thickness could be attributed to the re-passivation process of DMATMS. Although DMATMS is selective to SiO_2_/TiN patterns, it can lead to the formation of SiCO islands during re-passivation on the Ru surface [69]. These islands likely inhibit growth to some extent, thereby reducing the final expected thickness. However, despite this effect, we were still able to achieve our target thickness and decrease the Ru defects from 18% to ~8% Ru atomic concentration. The second approach involved etching the surface using O_2_/Cl_2_ plasma chemistry to remove defects after 40 ALD cycles. While a brief etching process effectively eliminates Ru defects, it also substantially reduces the thickness of the selectively grown film. A 7 s etch is conducted on DMATMS passivated SiO_2_/TiN surface after 40 Ru ALD cycles. The TEM, EDS spectral and elemental profiles are shown in Figure 8c,d,f. The Ru atomic concentration was reduced to less than 2% on SiO_2_ with a selective Ru thickness of 2.8 ± 0.5 nm remaining on the TiN bottom layer. Despite the defect etching, we lose ~1.5 nm of Ru film on the TiN growth area, and an 85% reduction in Ru defects on SiO_2_ is calculated. 

In summary, the proposed area-selective deposition (ASD) method can be utilized to selectively grow Ru hard mask and hold potential in a tone inversion patterning scheme. The DMATMS passivation employed in this study deposits on the surface relatively quickly compared to other larger inhibitors. A combination of ALD super cycles and etching holds the potential for achieving a larger selective Ru thickness on the growth area while simultaneously reducing the defects formed on the non-growth area. This can be achieved through a process involving passivation followed by ALD, subsequent etching, and repeating the cycle when necessary. The study also presents an opportunity for enhancing selectivity through the exploration of alternative surface passivation moieties, such as SMIs and SAMs, which can be applied using immersion, spin-coating or from the vapor phase. Additionally, an interesting avenue for improving Ru selectivity could involve investigating different co-reactants aside from oxygen, such as hydrogen.

## 4. Conclusions

Bottom–up patterning by ASD is an enabling manufacturing technique for device fabrication at critical dimensions at the nanometer scale, and defectivity is one of the main roadblocks for ASD. Ru is highly relevant for industrial applications because of better electrical performance in terms of resistivity and reliability than Cu.

In this work, a Ru ALD process using carbonyl-based Ru precursor and oxygen as a co-reactant is studied. This Ru deposition process shows a GPC as high as 1.6 Å/cycle, and Ru films can be deposited at temperatures as low as 220 °C. While the developed ALD process using 10 s oxygen co-reactant pulse time shows an acceptable Ru GPC on the growth area, its selectivity is marginal. By reducing the oxygen co-reactant pulse time from 10 s to 1 s, ALD selectivity is substantially improved for all the studied passivation layers on the NGA at the cost of a GPC reduction from 1.6 Å/cycle to 0.95 Å/cycle. Being defectivity the main issue for the ASD processes, this paper provides a detailed analysis of Ru particle size distribution and defect generation mechanisms which are correlated with the structure of different studied passivation layers on the NGA. 

When selecting a passivation layer, it is crucial to consider its structure in terms of functional groups in the backbone, layer density, and interaction between adjacent layers. These factors are paramount in reducing defects and achieving greater selectivity. DMATMS is found to be the best passivation layer, providing a selectivity of 0.9 for 30 ALD Ru cycles. In addition, DMATMS can selectively deposit on SiO_2_ with respect to TiN. Finally, the ASD of Ru films on 45 nm half-pitch SiO_2_/TiN patterns is demonstrated by combining the DMATMS organic layer passivation and ALD of Ru. A selective Ru film of ~4.4 nm on the TiN surface was measured after 40 ALD cycles, while the Ru atomic concentration on DMATMS passivated SiO_2_ was ~18% in DMATMS passivated SiO_2_. The selectivity was further improved by using two different approaches, namely, ALD super cycles and etching of Ru defects. In the case of ALD super cycles, which involve the DMATMS re-passivation after 10 ALD cycles for a total of 40 ALD cycles, a selective Ru film of ~3.4 nm is measured on the TiN surface with ~8% Ru atomic concentration in SiO_2_. In the second approach, a 7 s Ru etch is applied on the DMATMS passivated surface subjected to 40 Ru ALD cycles. A ~2.7 nm selective Ru film is observed on the TiN layer at the bottom of 45 nm half-pitch patterns with less than 2% atomic concentration of Ru on SiO_2_ sidewalls and top SiO_2_ surface.

## Figures and Tables

**Table 1:** ^a^—Bis(cyclopentadienyl)ruthenium(II), ^b^—Bis(ethylcyclopentadienyl)ruthenium(II), ^c^—Bis (η5-cycloheptadienyl)ruthenium(II), ^d^—Tris(2,2,6,6-tetramethyl-3,5-heptanedionato)ruthenium (III), ^e^—(Ethylbenzyl)(1-ethyl-1,4-cyclohexadienyl)ruthenium(0), ^f^—Bis(2,4-dimethylpentadienyl) ruthe nium(II), ^g^—ethylcyclopentadienyl pyrrolyl ruthenium, ^h^—(1,3-Butadiene)(ethylbenzene) ruthenium (0), ^i^—(1,3-Cyclohexadiene)(ethylbenzene)ruthenium(0), ^j^—η6-1-Isopropyl-4-methyl benzene(η4-cyclohexa-1,3-diene)ruthenium(0), ^k^—(1,5-Hexadiene)(1-isopropyl-4-methyl benzene) Ru(0), ^l^—Tri- carbonyl-(η4-2,3-dimethylbutadiene)ruthenium(0), ^m^—Bis(2,6,6-trimethyl-cyclohexadienyl)ruthe nium(II), ^n^—Bis(2,5-dimethylpyrrolyl)ruthenium(II), ^o^—Tricarbonyl(trimethylenemethane) ruthenium.

Precursor	Deposition Temp (°C)	Growth per Cycle (GPC) (Å/Cycle)	Incubation Cycle	Ref.
RuCp_2_^a^	275–400	0.45	<200	[38]
Ru(EtCp)_2_^b^	270	0.15	50	[39,40]
Ru(Chd)_2_^c^	200–300	0.2–0.4	~22	[41]
Ru(thd)_3_^d^	325–450	0.36	~200	[42]
EBECHRu ^e^	225–325	0.4	<10	[21,43]
Ru(DMPD)_2_^f^	165–250	0.12	~50	[44,45]
(EtCp)Ru(Py) ^g^	275–350	0.47	<150	[46]
EBBDRu ^h^	225	0.6	~15	[47]
EBCHDRu ^i^	140–350	1	~2	[37,48]
IMBCHDRu ^j^	225–270	0.89	~11	[49]
IMBHDRu ^k^	230–350	0.76	~3	[50]
Ru(DMBD)(CO)_3_^l^	290–320	0.67	0	[51,52]
Cyprus ^m^	250–300	0.5	~50	[53]
DMPR ^n^	275–300	0.55	~50	[54]
Ru(TMM)(CO)_3_^o^	200–260	1.5–1.7	6	[29,30] this work

## Data Availability

Data sets are available upon reasonable request.

## Supplementary Materials

The following supporting information can be downloaded at: https://www.mdpi.com/article/10.3390/nano14141212/s1, Figure S1: AFM of surface (a); pristine Si with native oxide (b); 10 Ru ALD cycles on Si; (c) 30 Ru ALD cycles on Si; (d) 50 Ru ALD cycles on Si; Figure S2: TD-SEM and X-SEM images of Ru ALD on Si surface after (a,e) 10 ALD cycles; (b,f) 30 ALD cycles; (c,g) 50 ALD cycles.; Figure S3: TD-SEM of Ru ALD on FDTMS passivated Si surface after (a) 5 ALD cycles; (b) 10 ALD cycles; (c) 20 ALD cycles; (d) 30 ALD cycles; (e) 50 ALD cycles.; Figure S4: SEM images of Ru ALD performed on FDTMS passivated surface at reduced oxygen pulse time of 0.5 s (a–d) and 1 s (e–f). Ru ALD cycles vary from 5–30 cycles; (a,e) 5 cycles; (b,f) 10 cycles; (c,g) 20 cycles; (d,h) 30 cycles.; Figure S5: SEM images of Ru defects generated on SAM passivated surface (a–d) liquid TMOS (e–h) vapor TMOS (i–l) DMATMS. The ALD is performed at different cycles (a,e,i) 5 ALD cycle (b,f,j) 10 ALD cycle (c,g,k) 20 ALD cycle (d,h,l) 30 ALD cycle; Figure S6: 20° tilted SEM images (a–d) and X-SEM images (e–h) for SiO_2_/TiN pattern passivated with DMATMS. Ru ALD of (a,e) 20 cycles; (b,f) 30 cycles; (c,g) 40 cycles; (d,h) 50 cycles were performed on this DMATMS passivated pattern.; Figure S7: Equivalent Ru thickness derived from RBS on pristine TiN surface assuming a Ru density of 12.36 g/cm^3^; Table S1: Ru areal density calculated on pristine Si and SAM, SMIs deposited Si surfaces.
