# Supplementary material for: Area-Selective Atomic Layer Deposition of Ru Using Carbonyl-Based Precursor and Oxygen Co-Reactant: Understanding Defect Formation Mechanisms
Source: Nanomaterials (Basel). 2024 Jul 16;14(14):1212. doi: 10.3390/nano14141212 (PMC11280396; doi:10.3390/nano14141212)
Supplement: Supplementary file 1 [file nanomaterials-14-01212-s001.zip › nanomaterials-3077367-supplementary.pdf]

# Area selective-atomic layer deposition of Ru using carbonyl-based precursor and oxygen co-reactant: understanding defect formation mechanisms

**Jayant K. Lodha [1,2], Johan Meersschaut [2], Mattia Pasquali [2], Hans Billington [2], Stefan De Gendt [1, 2], Silvia Armini [2, *]**

[1] Department of Chemistry, Faculty of Science, KU Leuven, B-3001 Leuven, Belgium

[2] Semiconductor Technology and System, Imec, Kapeldreef 75, B-3001 Leuven, Belgium

* Correspondence: silvia.armini@imec.be

## S1: AFM images after Ru ALD

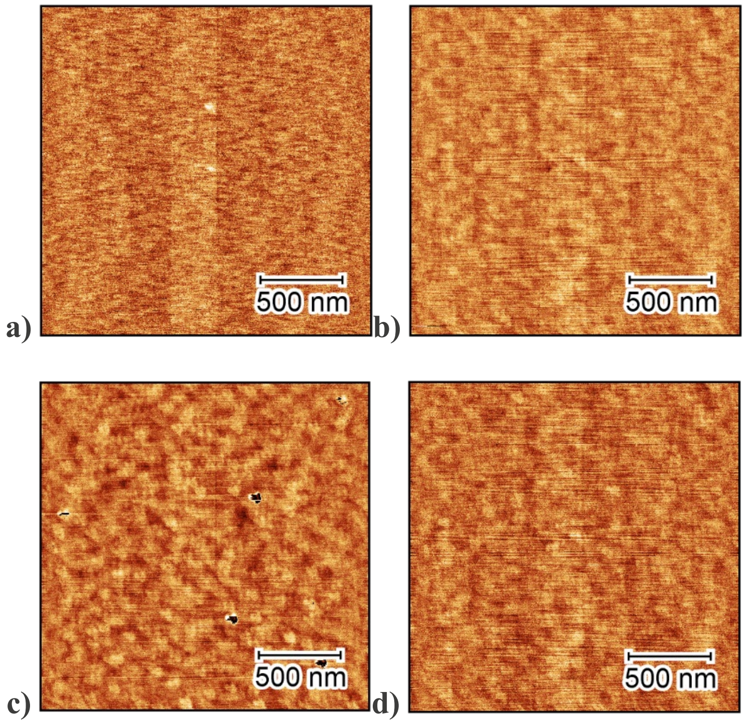


Figure S1: AFM of surface a) pristine Si with native oxide b) 10 Ru ALD cycle on Si c) 30 Ru ALD cycle on Si d) 50 Ru ALD cycle on Si

Atomic force microscopy (AFM) images are taken for pristine Si surface and after Ru ALD from 10-30 cycles. The roughness measured is below 0.1 nm for all the surface confirming a smooth Ru film deposited on Si surface.

## S2: TD-SEM and X-SEM images of Ru films after ALD

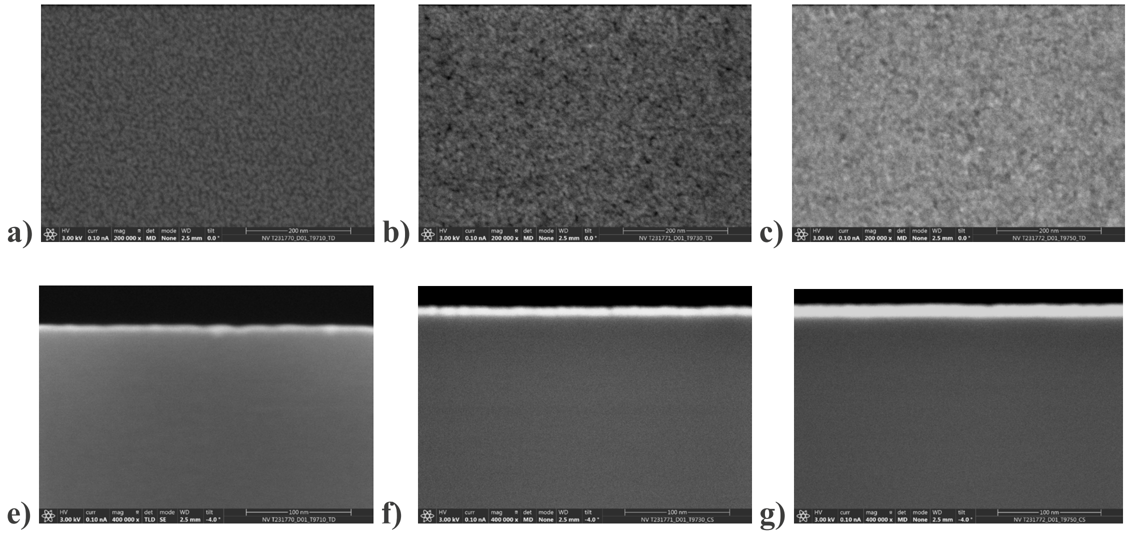


Figure S2: TD-SEM and X-SEM images of Ru ALD on Si surface after (a, e) 10 ALD cycle (b, f) 30 ALD cycle (c, g) 50 ALD cycle.

## S3: TD-SEM image of Ru defects on FDTMS passivated surface

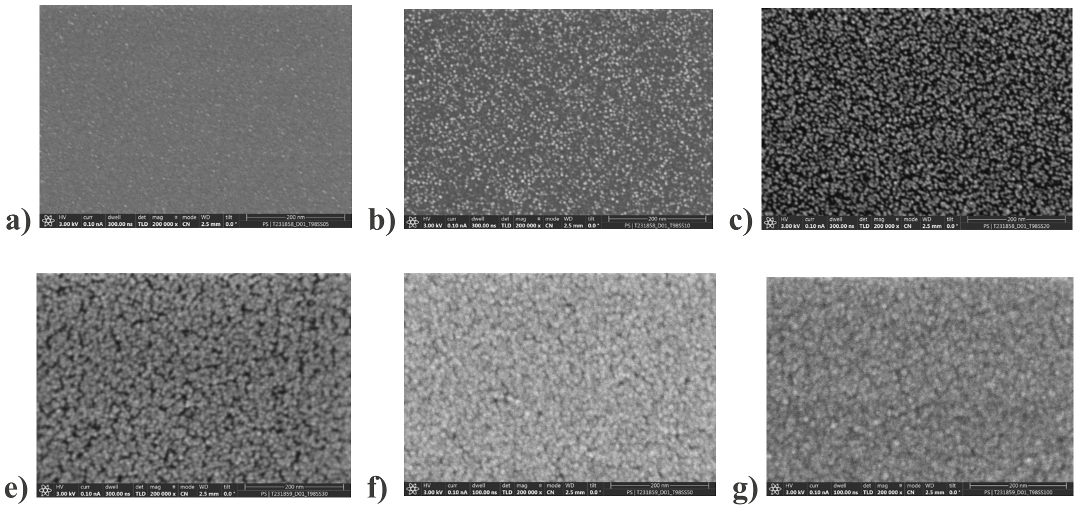


Figure S3: TD-SEM of Ru ALD on FDTMS passivated Si surface after a) 5 ALD cycle b) 10 ALD cycle c) 20 ALD cycle d) 30 ALD cycle e) 50 ALD cycle.

Ru ALD was performed on FDTMS passivated Si surface varying 5-50 ALD cycle. SEM images shows Ru defects generated already at 5 ALD cycle. The ALD condition here uses 11 s of Ru precursor pulse time and 10 s of oxygen co-reactant pulse time.

## S4: Comparison of Ru defects on reduced oxygen dosage by SEM image

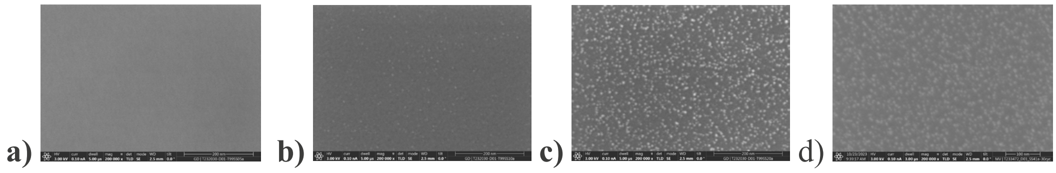

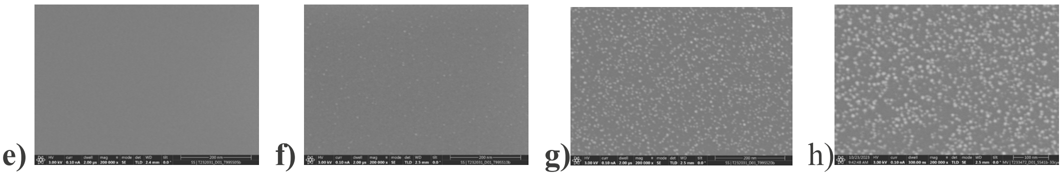

e) f) g) h)

Figure S4: SEM images of Ru ALD performed on FDTMS passivated surface at reduced oxygen pulse time of 0.5 s (a-d) and 1 s (e-f). Ru ALD cycles vary from 5-30 cycles (a, e) 5 cycle (b, f) 10 cycles (c, g) 20 cycles (d, h) 30 cycles.

## S5: Ru areal density calculated by RBS

Table S1: Ru areal density calculated on pristine Si and SAM, SMIs deposited Si surfaces.

| ALD cycle | Ru areal density ($10^{15}$ atm/cm$^2$) | | | | | |
|---|---|---|---|---|---|---|
| | Pristine $Si/SiO_2$ | TMODS | Liq. TMOS | Vap. TMOS | DMATMS | FDTMS |
| 5 | 0.38 | 0.03 | 0.03 | 0 | 0.01 | 0.02 |
| 10 | 3.15 | 0.03 | 0.62 | 0.21 | 0.04 | 0.06 |
| 20 | 14.22 | 0.03 | 6.97 | 3.77 | 0.35 | 0.8 |
| 30 | 25.57 | 0.03 | 19.42 | 10.64 | 1.7 | 2.76 |
| 40 | 33.43 | 0.03 | 29.79 | 16.94 | 2.92 | 9.27 |
| 50 | 47.23 | 0.02 | 40 | 33.41 | 6.48 | 36.1 |

## S6: SEM image for Ru defects on DMATMS, TMOS by liquid and vapor phase

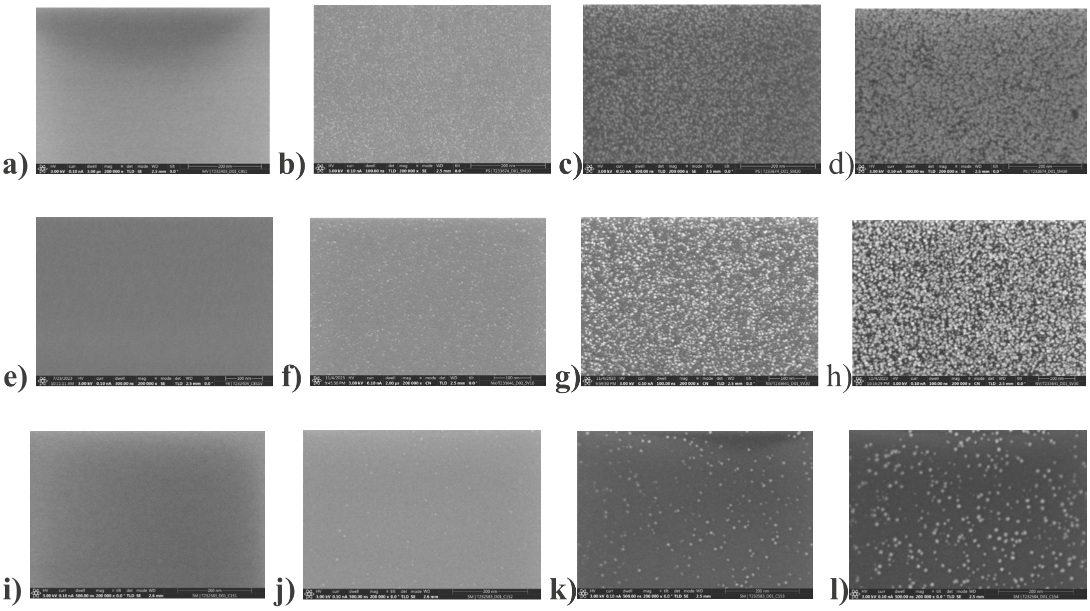

Figure S5: SEM images of Ru defects generated on SAM passivated surface (a-d) liquid TMOS (e-h) vapor TMOS (i-l) DMATMS. The ALD is performed at different cycles (a, e, i) 5 ALD cycle (b, f, j) 10 ALD cycle (c, g, k) 20 ALD cycle (d, h, l) 30 ALD cycle.

Ru ALD process here uses 5 s of Ru precursor pulse time and 1 s of oxygen co-reactant at 220°C. The optimized ALD condition is used to check selectivity against all organic layer (TMODS, TMOS, DMATMS, FDTMS). The selectivity seen in DMATMS is 0.9 with 30 ALD cycles.

## S7: Cross-sectional and tilted SEM images of $SiO_2$/TiN pattern

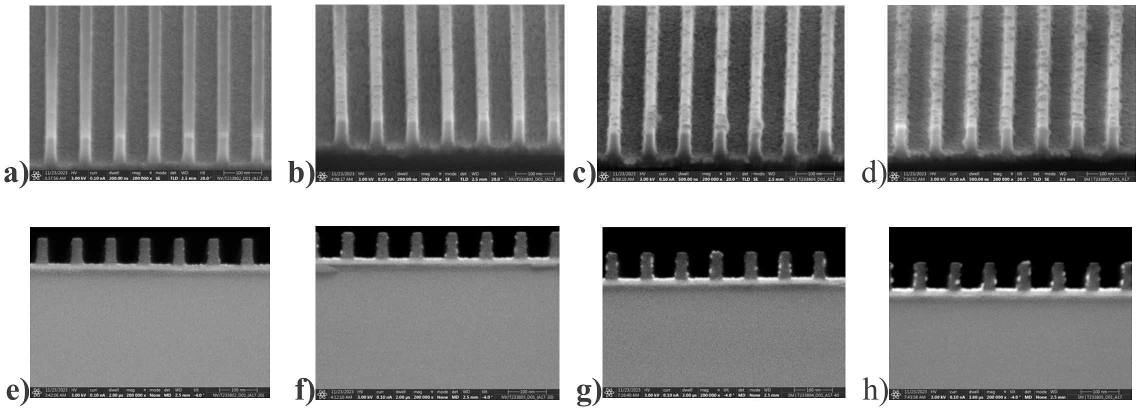

Figure S6: 20° tilted SEM images (a-d) and X-SEM images (e-h) for $SiO_2$/TiN pattern passivated with DMATMS. Ru ALD of (a, e) 20 cycles (b, f) 30 cycles (c, g) 40 cycles (d, h) 50 cycles were performed on this DMATMS passivated pattern.

## S8: Ru growth on blanket TiN surface by RBS

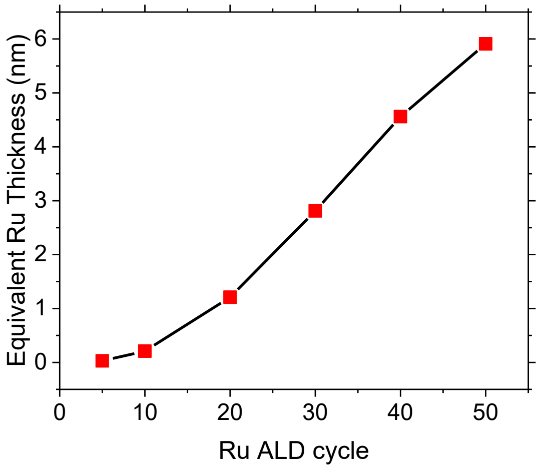


Figure S7: Equivalent Ru thickness derived from RBS on pristine TiN surface assuming a Ru density of 12.36 gm/cm$^3$.
